# Marine Cyanobacteria: A Source of Lead Compounds and their Clinically-Relevant Molecular Targets

**DOI:** 10.3390/molecules25092197

**Published:** 2020-05-08

**Authors:** Lik Tong Tan, Ma Yadanar Phyo

**Affiliations:** Natural Sciences and Science Education, National Institute of Education, Nanyang Technological University, Singapore 637616, Singapore; zaara.yadanarphyo@gmail.com

**Keywords:** marine cyanobacteria, natural products, drug discovery, molecular targets

## Abstract

The prokaryotic filamentous marine cyanobacteria are photosynthetic microbes that are found in diverse marine habitats, ranging from epiphytic to endolithic communities. Their successful colonization in nature is largely attributed to genetic diversity as well as the production of ecologically important natural products. These cyanobacterial natural products are also a source of potential drug leads for the development of therapeutic agents used in the treatment of diseases, such as cancer, parasitic infections and inflammation. Major sources of these biomedically important natural compounds are found predominately from marine cyanobacterial orders Oscillatoriales, Nostocales, Chroococcales and Synechococcales. Moreover, technological advances in genomic and metabolomics approaches, such as mass spectrometry and NMR spectroscopy, revealed that marine cyanobacteria are a treasure trove of structurally unique natural products. The high potency of a number of natural products are due to their specific interference with validated drug targets, such as proteasomes, proteases, histone deacetylases, microtubules, actin filaments and membrane receptors/channels. In this review, the chemistry and biology of selected potent cyanobacterial compounds as well as their synthetic analogues are presented based on their molecular targets. These molecules are discussed to reflect current research trends in drug discovery from marine cyanobacterial natural products.

## 1. Introduction

The photosynthetic filamentous marine cyanobacteria are prokaryotic microorganisms found in diverse habitats, including epiphytic, epilithic, endolithic and microbial mats communities, in coral reef ecosystems [1]. Natural products research on marine cyanobacteria has revealed its impressive biosynthetic capacity in producing structurally novel bioactive secondary metabolites [2]. The high success rate of marine cyanobacteria in colonizing different aquatic habitats could be attributed to the ecological roles of these compounds, such as UV-radiation protection, feeding deterrence, allelopathy and signaling [3]. To date, more than 550 secondary metabolites have been reported from diverse marine cyanobacterial genera, including *Lyngbya*, *Moorea*, *Symploca* and *Oscillatoria* [4]. A majority of these biomolecules are nitrogen-containing and are products of the modular biosynthetic enzymes, such as the non-ribosomal peptide synthetases (NRPS), polyketide synthases (PKS) and hybrid NRPS-PKS [5]. Moreover, research on the biosynthetic machinery of these microbial systems revealed unusual mechanistic and enzymatic features, resulting in the production of diverse chemical structures [5].

Several pharmacological trends have been observed amongst the various marine cyanobacterial secondary metabolites. A significant number of molecules have been reported to possess either potent cytotoxic (e.g., largazole, coibamide A and curacin A), neuromodulating (e.g., antillatoxin, kalkitoxin and jamaicamides) or antiinfective (e.g., almiramides and gallinamide A) properties [6,7,8,9]. The high potency of these compounds is due to their specific disruption/interference with validated drug targets implicated in various human diseases, including cancer, inflammation and neurodegenerative disorders. These drug targets include enzymes, e.g., proteasomes, proteases, and histone deacetylases, cellular cytoskeletal structures, e.g., microtubules and actin filaments, as well as membrane channels, e.g., voltage-gated sodium channels and Sec61 protein translocation channels. As such, these natural products make excellent lead compounds for drug discovery and development. For instance, a number of marine cyanobacterial compounds and their synthetic analogues formulated as Antibody-Drug Conjugates (ADCs), including dolastatin 10, auristatin E and OKI-179, have undergone/undergoing clinical trials for the treatment of cancer diseases [10,11].

Integrated genomic and metabolomics approaches have been used to mine marine cyanobacteria for structurally unique natural products. In particular, the MS/MS-based metabolomics platform, Global Natural Product Social Molecular Networking (GNPS), is a powerful tool for rapid chemical profiling of natural products mixtures [12]. The use of GNPS, in combination with genomic or biological assays, has extended the accessible chemical space for the discovery of novel bioactive cyanobacterial compounds. For instance, a new class of acyl amides, columbamides, with cannabinomimetic activity was uncovered based on genomic and mass spectrometric profiling of three marine cyanobacterial strains of the genus *Moorea* [13]. In addition, the use of bioassay-guided fractionation and MS-based molecular networking resulted in the isolation of a cytotoxic cyclic octapeptide, samoamide A [14]. Moreover, recent advances in NMR spectroscopy have enabled new strategies for detection of new natural products from cyanobacterial extracts. One such tool is the recently developed Small Molecule Accurate Recognition Technology (SMART), which is based on a convolutional neural network to classify 2D NMR spectra, such as HSQC spectra, of natural products using pattern recognition principles [15]. Such innovative NMR-based method has led to the detection of several new cyclic depsipeptides belonging to the viequeamide class of molecules as well as new chimeric swinholide-like macrolide, symplocolide A [16,17].

Due to the sheer number of reported bioactive marine cyanobacterial compounds, it is probably impossible to provide a comprehensive coverage of all potent compounds from marine cyanobacteria in this review. There are a number of reviews on the diversity of marine cyanobacterial compounds as well as their pharmaceutical importance that readers can refer to [2,18,19,20,21,22,23,24,25]. Instead, this mini review will feature selected potent natural products and their clinically relevant molecular targets, including both enzyme and non-enzyme-based targets/pathways. The selection includes cyanobacterial molecules that have been identified as drug leads for further structural optimization as well as SAR studies. These natural products and their synthetic analogues are discussed based on their interference with molecular targets/pathways, such as histone deacetylases, proteasomes, proteases, actin and microtubule filaments, and membrane receptors/channels. Furthermore, this review serves to complement and provide an update on information of two reviews previously published by Tan as well as Salvador-Reyes and Luesch [21,22].

## 2. Marine Cyanobacterial Drug Leads and their Molecular Targets

### 2.1. Histone Deacetylases

#### 2.1.1. Largazole

**Figure 1:**
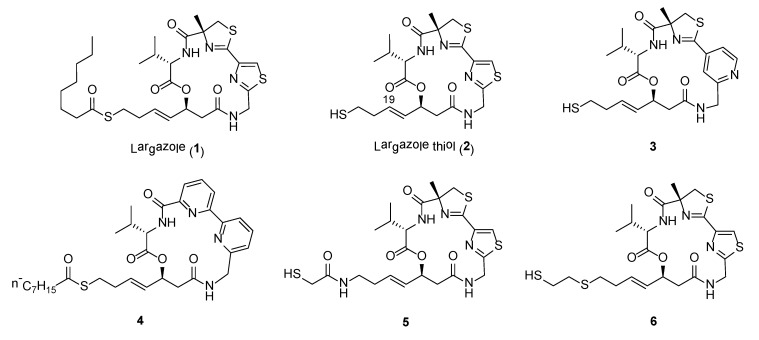
Largazole and related compounds.

Largazole (**1**) (Figure 1), a cyclic depsipeptide, is a highly potent class I histone deacetylase (HDAC) inhibitor originally discovered from the marine cyanobacterium, *Symploca* sp., collected from Key Largo (FL, USA) [26]. Largazole consists of a number of unusual structural features, including a 3-hydroxy-7-mercaptohept-4-enoic acid unit and the linkage of a 4-methylthiazoline unit to a thiazole. The compound is found to be a potent inhibitor on the growth of transformed human mammary epithelial cells (MDA-MB-231) with GI_50_ of 7.7 nM. In addition, compound **1** showed exquisite antiproliferative activity against transformed fibroblastic osteosarcoma U2OS cells (GI_50_ 55 nM) over non-transformed fibroblasts NIH3T3 (GI_50_ 480 nM) when compared to paclitaxel, actinomycin D, and doxorubicin [26].

Largazole is a prodrug and upon hydrolysis of the thioester provides the highly active largazole thiol (**2**) (Figure 1) [27]. Due to the impressive anticancer property and unique structure of largazole, it has attracted interest from synthetic chemists on its total synthesis and generation of synthetic analogues. To date, more than a dozen reports on its total synthesis have been successfully accomplished [28]. A scale-up synthesis of largazole was also recently reported by Chen et al., where the syntheses of each fragment and final product were optimized to achieve an overall yield of 21% in eight steps [29]. In addition, various in vivo and in vitro biological as well as epigenetic studies performed on largazole revealed its potential use in broad spectrum therapy (Table 1) [30,31,32,33,34,35,36,37,38,39,40,41].

Extensive SAR studies on synthetic largazole-based analogues were carried out and the results of these studies were summarized recently by Poli et al. [42]. In general, the thiazole-thiazoline unit of largazole is the recommended moiety where structural modifications should be carried out to generate new analogues with selective inhibition on HDAC as well as anticancer activity. For instance, pyridine analogues (e.g., **3**) and bipyridine analogue, **4**, provided potent anticancer and HDAC inhibitory activities (Figure 1, Table 2) [43,44]. Modification to the L-valine unit in largazole is well tolerated and in some cases able to confer higher selectivity for cancer cells over normal cells. In addition, changes to the conformation and the flexibility of the macrocyclic core can impact HDAC activity as well as on the selectivity for HDACs 1-3 over HDAC6 [45]. Furthermore, largazole analogues with modified warheads generally showed decreased in activity. However, a number of analogues containing zinc binding groups, including α-thioacetamide (e.g., **5**) or mercaptosulfide (e.g., **6**), have altered selectivity profile, such as conferring selectivity for HDAC6 or HDAC10 (Figure 1, Table 2) [46,47]. Largazole analogues with selectivity for HDAC6 or HDAC10 have possible usage for the treatment of CNS disorders or ovarian cancer, respectively [48,49]. Recent study on the incorporation of monofluoro and gem-difluoro substitution at the Zn^2+^-binding thiolate side chain of largazole thiol **2** revealed that C19-position fluoro-substituted largazole analogue exhibited high selectivity against HDAC1 over HDAC6 [50]. Due to the exceptional activity of largazole, new studies on the design of synthetic analogues with enhanced potency and selectivity are expected to be pursued to expand the chemical space of this class of compounds.

Amongst the various largazole analogues, a synthetic compound, OKI-179, has recently been identified as a drug candidate and subsequently, proceeded to Phase I clinical trial for the treatment of advanced solid tumors [51,52]. OKI-179 was developed based on several chemical optimization steps involving largazole thiol and OKI-006. OKI-179 showed potent inhibition of the Class 1 HDACs, namely HDAC 1, 2 and 3, with IC_50_s’ values of 1.2, 2.4 and 2.0 nM, respectively. Moreover, it shows impressive antiproliferative and apoptotic properties against a wide range of cancer cell lines, activity in xenograft cancer models as well as good oral pharmacokinetic properties in mouse, rat and dog models. Results from the first dosing cohorts revealed that the drug was well-tolerated and achieved good exposure following oral administration [52].

#### 2.1.2. Santacruzamate A

**Figure 2:**
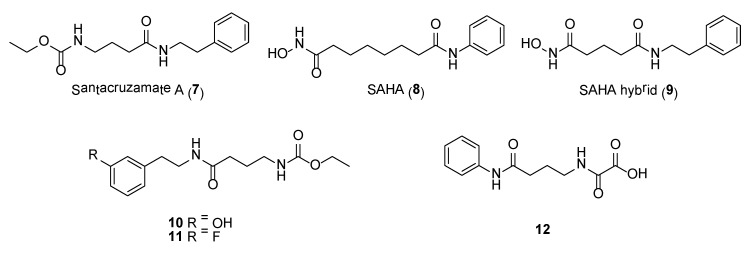
Santacruzamate A, SAHA and related compounds.

A picomolar-range histone deacetylase inhibitor, santacruzamate A (**7**) (Figure 2), was obtained from the organic extracts of a tuft-forming marine cyanobacterium collected from Coiba National Park, Panama [53]. Due to the structural similarities of santacruzamate A with the clinically approved HDAC inhibitor, suberoylanilide hydroxamic acid (= SAHA, **8**) (Figure 2), the natural product was further evaluated in a series of anti-HDAC assays. This led to the discovery of santacruzamate A as a potent inhibitor of HDAC2, a Class I HDAC, with an IC_50_ of 119 pM. Compound **7** also showed cytotoxicity against HCT116 and Hut-78 cancer cell lines with GI_50_ of 28.3 µM and 1.4 µM, respectively. Total synthesis, achieved in two steps, of santacruzamate A as well as a synthetic hybrid compound, **9** (Figure 2), was reported [54]. 

Synthetic compound **9** was found to be 30 times less potent compared to santacruzamate A when tested against HDAC2. In another SAR study by Randino et al., two classes of santacruzamate derivatives having several aromatic amines containing either the ethyl carbamate unit (class I) or oxamic acid moiety (class II) as zinc-binding groups were synthesized [55]. The study identified three synthetic molecules, including **10**, **11** and **12** (Figure 2), having potent antiproliferative activity in HCT116 cancer cells, with IC_50_s’ of 0.476 µM, 0.825 µM and 0.814 µM, respectively [55]. However, HDAC inhibitory activity was not detected in these synthetic derivatives, which underscore the importance of the zinc binding group positioning into the HDAC catalytic site as a crucial step in the inhibition process.

Recent pharmacological study using santacruzamate A, in combination with other HDAC inhibitors, such as HDAC1 inhibitor, tacedinaline, HDAC1/2 common inhibitor, romidepsin (FK228) and global HDAC inhibitor, vorinostat (SAHA), to treat hepatocellular carcinoma (HCC) was reported [56]. The combined chemotherapy led to the inhibition of HDAC1/2 as well as changes in HCC cell morphology, growth inhibition, cell cycle blockage and apoptosis in vitro and growth suppression of subcutaneous HCC xenograft tumors in vivo [56]. Santacruzamate A has also been explored as potential candidate lead compound for the treatment of endoplasmic reticulum (ER) stress- and unfolded protein response (UPR)-related neurodegenerative disorders, such as Alzheimer’s disease [57].

### 2.2. Proteasome

#### Carmaphycins

**Figure 3:**
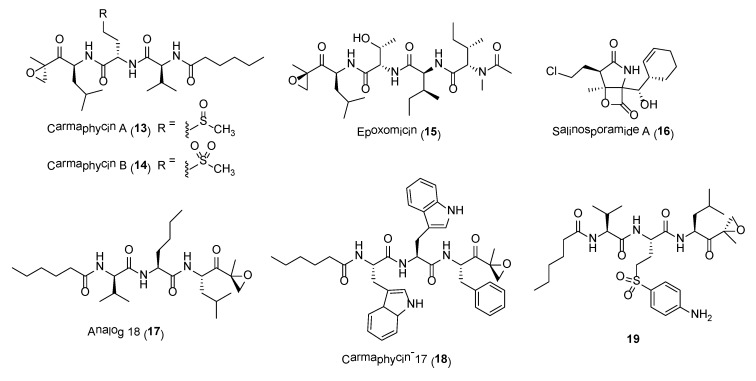
Carmaphycins, epoxomicin, salinosporamide A and related compounds.

Carmaphycins A (**13**) and B (**14**) (Figure 3) are potent novel proteasome inhibitors isolated in low yield from organic extracts of *Symploca* sp. obtained from CARMABI beach, Curacao [50]. Structurally, the carmaphycins consist of a leucine-derived α,β-epoxyketone warhead directly attached to either a methionine sulfoxide in **13** or a methionine sulfone in **14**, which is linked to a valine and an alkyl chain terminal tail. Their total synthesis was accomplished using an efficient and scalable convergent method [58]. The proteasome inhibitory properties of carmaphycins A and B were evaluated against *Saccharomyces cerevisiae* 20S proteasome and found to have comparable IC_50_ values of 2.5 nM and 2.6 nM, respectively. Such inhibitory activities are comparable with those reported for epoxomicin (**15**) and the marine-derived salinosporamide A (**16**), with IC_50_ values of 2.7 nM and 1.4 nM, respectively (Figure 3). In addition, cytotoxic assay showed carmaphycins to be particularly active against solid tumor cell lines, including human lung adenocarcinoma and colon cancer cell lines. Preliminary structural biology investigation of the carmaphycins suggested distinct binding site as compared to epoxomicin, salinosporamide A and bortezomib.

**Scheme 1:**
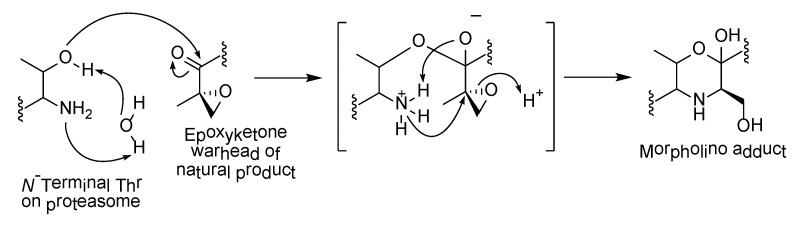
Mechanism of action of carmaphycins with α,β-epoxyketone warhead.

The α,β-epoxyketone warhead in carmaphycins is also a structural feature in epoxomicin (**12**), a known proteasome inhibitor, as well as the FDA-approved anticancer drug, carfilzomib. Their irreversible inhibition of proteasome is due to the formation of the morpholino adduct between the epoxyketone warheads with the Thr1 residues in the catalytic sites of the 20S proteasome core. The mechanism involves warhead carbonyl and epoxide undergoing two successive nucleophilic attacks by Thr1 Oγ and Thr1 N, respectively (Scheme 1) [59]. A novel mechanism of proteasome inhibition via hydroamination using alkene derivatives of the carmaphycin was recently reported by Trivella and co-workers [60]. The action of the carmaphycin enone electrophile was found to be partially reversible and this could provide further insights on the design of proteasome inhibitors for cancer treatment.

A number of synthetic analogues of carmaphycin B have been explored as lead molecules for the treatment of protozoan and metazoan parasitic infection as well as cancer. One such synthetic compound is analog 18 (**17**) (Figure 3), which was synthesized based on the structure of carmaphycin B [61]. Analog 18 was shown to possess potent in vitro antimalarial activity against asexual blood stages and gametocytes as well as strongly inhibits the activity of *Plasmodium* proteasome. Its potent activity was due to specific inhibition of the β5 subunit of the proteasome. Moreover, the study showed that incorporating a d-amino acid at the P3 position can significantly modulate host cytotoxicity without interfering significantly with anti-plasmodial activity [61]. In another study, a carmaphycin-related synthetic analogue, carmaphycin-17 (**18**, Figure 3), was found to have greater inhibitory activity against *Trichomonas vaginalis* than the reference drug metronidazole [62]. *Trichomonas vaginalis* is the causative agent of the sexually transmitted disease, trichomoniasis. In addition, **18** was found to be selective for *T. vaginalis* due to its increased potency against the β1 and β5 catalytic subunits of the *T. vaginalis* proteasome as compared to the human proteasome subunits [62]. Compound **18** is also shown to be selective for the *Schistosoma mansoni* proteasome (about 13.2-fold more potent) over the human proteasome [63]. Treatment of adult *S. mansoni* with **18** resulted in the same phenotypic changes as the FDA-approved drugs, bortezomib and carfilzomib.

In another development, a library of carmaphycin B analogues containing amine handles were synthesized and screened for their cytotoxic properties as Antibody-Drug Conjugates [64]. The screening effort resulted in identifying a highly potent analog of carmaphycin B, **19** (Figure 3), containing a 4-sulfonylaniline handle as an attachment point for the linker antibody. However, the study found that the free analogues, such as **19**, gave better cytotoxic activity at sub-nanomolar levels as compared to their ADCs when tested against the SKBR3 and MDA-MB-231 cancer cell lines. In addition, it was revealed that the cytotoxicity of free analogues with linear amines was greatly reduced as compared to free analogues with aromatic amines. The reduced potency of the ADCs could be due to protein-mediated degradation of the conjugated carmaphycin backbone, lysosomal degradation of the analogues or inappropriate ADC trafficking [64].

### 2.3. Protease Enzymes

Proteases are ubiquitous proteolytic enzymes found in eukaryotic and prokaryotic cells. The classification of these enzymes is based on key catalytic group present in the active site, such as serine, threonine, cysteine, aspartate, glutamate or zinc in metalloproteases. They account for about 2% of the genes in human cells and are involved primarily in proteins activation, synthesis and turnover [65]. Due to their involvement in many signaling pathways, they represent potential drug targets in human diseases, including cardiovascular disorders, inflammation, cancers as well as parasitic and viral infections [66]. It has been reported that filamentous marine cyanobacteria are sources of potent protease inhibitors, particular molecules targeting serine and cysteine proteases.

#### 2.3.1. Serine Protease Inhibitors

Serine proteases, including elastase, chymotrypsin, and trypsin, are a large class of enzymes having different roles related to human health, such as immune response, wound healing and blood coagulation. Studies have shown that an increase or decrease of protease activity can induce pathologies, including cancer, inflammation, heart attack, stroke and pancreatitis [67]. A number of potent serine protease inhibitors have been reported from marine cyanobacteria. 

**Figure 4:**
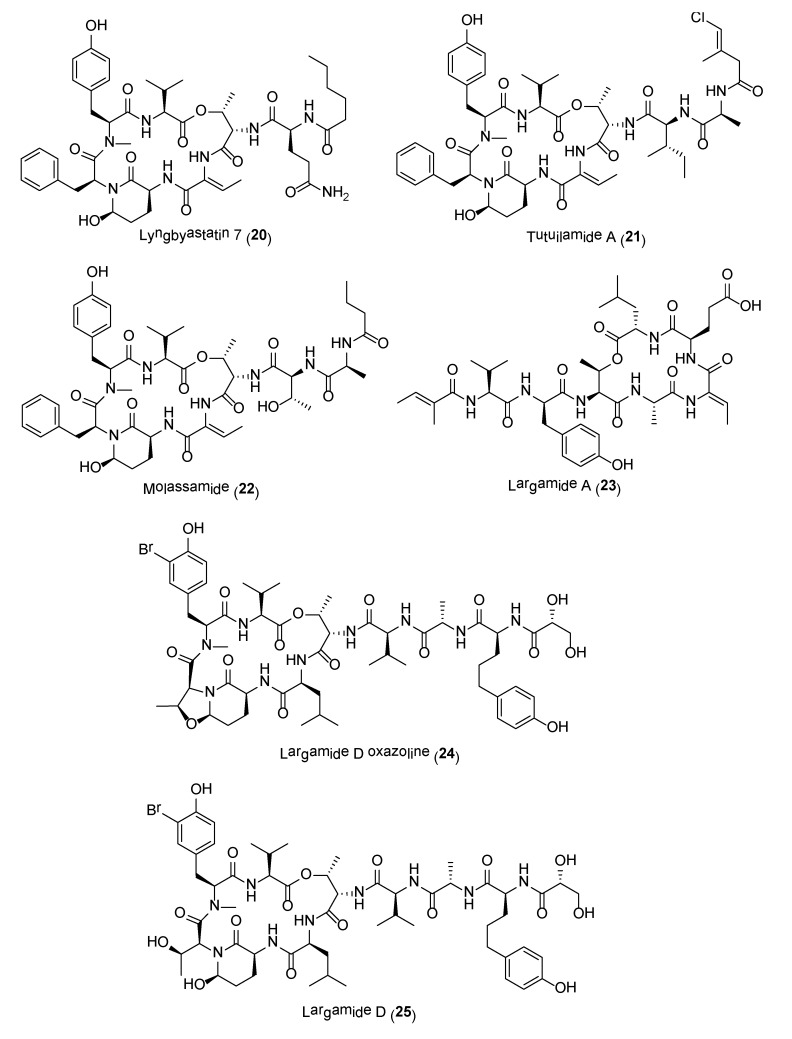
Serine protease inhibitors from marine cyanobacteria.

A majority of these serine protease inhibitors are 3-amino-6-hydroxypiperidone (Ahp)-containing cyclodepsipeptides, including lyngbyastatins 4–10 (e.g., **20**) (Figure 4), pompanopeptin A, symplocamide A, kempopeptins, molassamide, bouillomides, somamide B as well as recently reported molecules, loggerpeptins, kyanamide and tutuilamides (e.g., **21**) [68,69,70,71,72,73,74,75,76,77,78]. All Ahp-cyclodepsipeptides contain an N-methyl aromatic amino acid at conserved positions in their 19-membered ring structure, while other residues are less conserved. In addition to elastase selectivity, molassamide (**22**) was reported to inhibit the migration of the highly invasive MDA-MB-231 breast cancer cells [76]. Other non Ahp-containing cyclodepsipeptides, such as the largamides (e.g., **23**) and tiglicamides, have also been reported to possess moderate to potent serine protease inhibitory activities (Figure 4) [79,80,81].

It has been proposed that these cyanobacterial serine protease inhibitors may function as chemical defenses against marine predators [82]. Ecological studies by Matthew and co-workers led to the isolation of an Ahp-containing largamide D derivative **24** (Figure 4), formed via intramolecular condensation of largamide D (**25**) (Figure 4). This molecule, largamide D oxazolidine (24), exhibited 11-fold and 33-fold reduction in activity against chymotrypsin and elastase, respectively, when compared to largamide D [82]. The Ahp moiety is essential for serine protease inhibition and any structural or conformational changes to this unit will affect activity.

**Figure 5:**
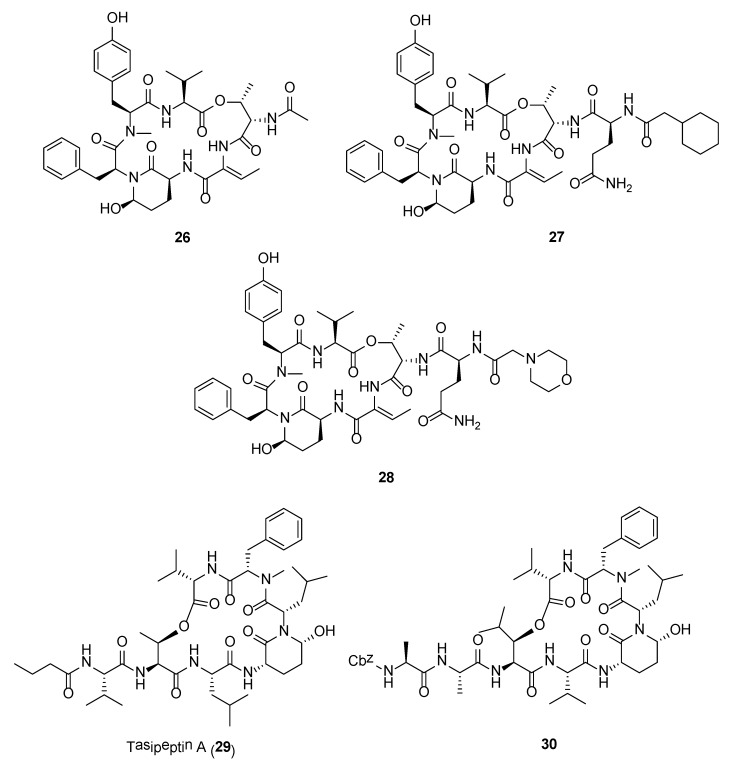
Lyngbyastatin 7 synthetic analogues, tasipeptin A and related compound.

Crystal structures of Ahp-cyclodepsipeptides in complex with serine proteases revealed that the inhibition is based on a substrate-like binding mode where distinct amino acid residues sit in the S- and S′-pockets with no proteolytic cleavage. These cyanobacterial inhibitors act in a similar manner to proteinaceous canonical serine protease inhibitors, where the conserved residues stabilize the inhibitory fold, and the other less conserved residues define serine protease selectivity through optimal accommodation at the specific S and S′-sites [83]. Such finding suggests that Ahp-cyclodepsipeptides may represent a suitable scaffold for designing non-covalent S1 serine protease inhibitors. As such a number of potent Ahp-cyclodepsipeptides has been targeted for total synthesis as well as generating synthetic analogues with enhanced activity. For instance, the total synthesis of lyngbyastatin 7 (**20**) was carried out in 31 steps by Luo and co-workers [84]. The synthetic compound showed superior activity over the only approved elastase inhibitor drug, sivelestat. Specifically, compound **20** demonstrated strong ability in protecting bronchial epithelial cells against elastase-induced antiproliferation as well as negating the elastase-triggered induction of pro-inflammatory cytokine expression [84]. A scale-up synthesis of lyngbyastatin **7** was recently developed with improved reaction yields and simplified purification steps [85]. Based on this synthetic strategy, a pilot library of lyngbyastatin 7 analogues (e.g., 26–28) was constructed for the purpose of compound tailoring and modulation of lipophilicity/hydrophilicity via manipulation of the pendent side chain. More importantly, this study revealed the importance of the side chain for elastase inhibition in addition to the Ahp and 2-aminobutenoic acid moiety in the core cyclic structure of lyngbyastatin 7. This major development would path the way for the translation of the natural product into pharmacotherapeutics for the treatment of diseases with overactive human neutrophil elastase [85]. In another study, a practical mixed solid- and solution-phase synthesis were employed for the synthesis of tasipeptins (e.g., **29**) and synthetic analogues [86]. More importantly, this synthetic strategy resulted in the generation of the human HTRA protease inhibitors (e.g., **30**) with enhanced potency (Figure 5).

#### 2.3.2. Falcipain Inhibitors

##### Gallinamide A

**Figure 6:**
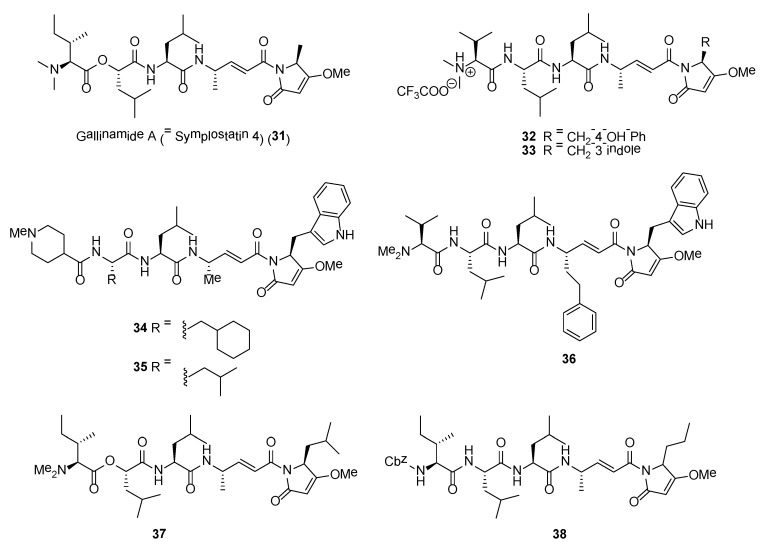
Gallinamide A and related compounds.

Gallinamide A (= symplostatin 4) (31) (Figure 6) is one of the most potent marine cyanobacterial antimalarial compounds reported to date, with EC_50_ of 74 nM when tested against *Plasmodium falciparum* strain 3D7 [87,88,89]. However, gallinamide A has moderate activity against mammalian Vero cells and no detectable cytotoxicity against the NCI-H460 lung tumor and neuro-2a mouse neuroblastoma cell lines [87]. Gallinamide A, a linear depsipeptide, has several unique structural features, including a dimethylated N-terminal amino acid moiety, a 4-amino-2-pentenoic acid unit, and a C-terminal *N*-acyl-pyrrolinone unit. Gallinamide A was initially reported by Linington and co-workers from an organic extract of a *Schizothrix* species collected from reef near Piedras Gallinas, Panama [87]. At the same time, symplostatin 4, having identical planar structure with gallinamide A, was also reported by Taori and co-workers from a different cyanobacterial species, *Symploca* sp., obtained from Florida Keys [88]. Based on the detailed 1D NMR analysis of the synthetic molecules, it was concluded that the structures of gallinamide A and symplostatin 4 are identical [89,90].

The potent antimalarial activity of gallinamide A (31) led to investigations by Stolze and co-workers on its mode of action and SAR studies based on synthetic and biochemical methods [91]. It was observed that *Plasmodium falciparum*-infected red blood cells treated with gallinamide A, at nanomolar concentration, exhibited swollen food vacuole phenotype. Through the use of fluorescent probes based on rhodamine fluorophore-tagged molecules, it was revealed that the natural product is a specific inhibitor of the plasmodial cysteine proteases, falcipains 2, 2′ and 3. In addition, the methoxypyrrolinone unit in gallinamide A is essential for antimalarial activity and modifications to the N-terminal end of the molecule are tolerated [91].

**Scheme 2:**
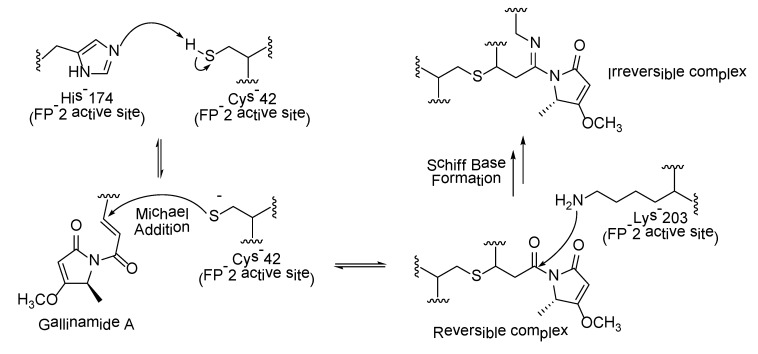
Proposed mechanism of gallinamide A at active site of falcipain 2 (FP-2) (adapted from Ref. [93]).

Falcipains are *Plasmodium falciparum* cysteine proteases involved in several key processes of the erythrocytic cycle of the malarial parasite, such as the hydrolysis of host hemoglobin, erythrocyte invasion, and rupture [92]. These cysteine proteases therefore constitute promising molecular targets in the search for novel antimalarial agents. The proposed mechanism of gallinamide A inhibition of falcipain 2 was deduced based on molecular dynamics simulation studies [93]. The simulation studies suggested that the methyl methoxylpyrrolinone moiety in gallinamide A inserts into the active site of falcipain 2, facilitating Michael addition and Schiff base formation with Cys-42 and Lys-203, respectively, leading to the irreversible inhibition of the enzyme (Scheme 2) [93].

A number of synthetic analogues, based on gallinamide A structural motif, having potent antiprotozoal or anticancer activity have been identified. In a study by Conroy and co-workers, analogues of gallinamide A were synthesized for SAR studies and tested for their inhibitory activity against falcipains 2 and 3 [94]. It was revealed that the α,β-unsaturated imide moiety of gallinamide A is important for inhibitory activity. In addition, several analogues, such as compounds **32** and **33** (Figure 6), showed potent inhibition of the chloroquine-sensitive 3D7 strain of *P. falciparum* as well as the chloroquine-resistant Dd2 strain of *P. falciparum* (Table 3) [94]. A recent study by Stoye et al. created a library of gallinamide A-based analogues using a highly efficient and convergent synthetic route [95]. Based on this library, several synthetic analogues (e.g., **34–36**) possessed potent inhibitory activity against the *P. falciparum* falcipain 2 and falcipain 3 as well as cultured chloroquine-sensitive (3D7) and chloroquine-resistant (W2) strains of *P. falciparum* (Table 3). Three synthetic lead molecules, namely **34, 35** and **36**, were subsequently evaluated for their in vivo efficacy against *P. berghei* infection in mice (Figure 6). Of the three compounds, compound **36** cured *P. berghei*-infected mice in the Peters 4 day-suppressive test when administered 25 mg kg^−1^ intraperitoneally daily over 4 days [95].

Synthetic analogues of gallinamide A were recently explored as potent inhibitor of cathepsin L for the treatment of Chagas disease [96]. The study, conducted by Boudreau and co-workers, showed that gallinamide A and its synthetic analogs potently inhibit cruzain, a *Trypanosoma cruzi* cysteine protease, and are exquisitely toxic toward *T. cruzi* in the intracellular amastigote stage. Compound 37 (Figure 6) was the most active cruzain inhibitor with an IC_50_ of 5.1 nM. This molecule was found to be inactive to the epimastigote and the host cell [96]. Synthetic analogues of gallinamide A were also explored for their anticancer activity. In a study by Liu et al., an anti-cancer stem cells lead molecule, **38**, was found to significantly suppress tumor growth both in vitro and *in vivo*. Moreover, **38** was able to significantly reduce the number of melanoma tumor spheres and decrease the percentage of ALDH+ melanoma cells by blocking the Wnt/β-catenin signaling pathway [97].

#### 2.3.3. Cathepsin Inhibitors

Cathepsins are protease enzymes, classified by various families, including serine protease, cysteine protease and aspartyl protease. There are currently about 15 classes of cathepsins in humans. These enzymes are essential for diverse range of normal physiological functions, including digestion, blood coagulation, bone resorption, ion channel activity, innate immunity, vesicular trafficking and autophagy. However, dysregulated cathepsins have been implicated in a number of pathologies, including arthritis, periodontitis, pancreatitis, macular degeneration, muscular dystrophy, atherosclerosis, obesity, stroke, Alzheimer’s disease, schizophrenia, tuberculosis, and carcinogenesis [98]. As such, cathepsins have a diagnostic value and are promising drug targets for a variety of human diseases through the inhibition of these proteases [99].

**Figure 7:**
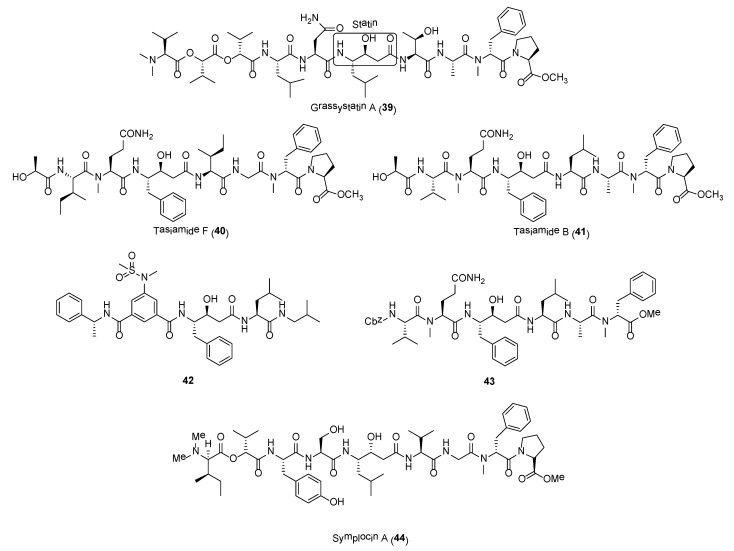
Grassystatin A, tasiamides B and F, symplocin A and related compounds.

A series of statine-containing linear depsipeptides, including grassystatins (e.g., **39**), tasiamides, and symplocin A, were found to be potent inhibitors of aspartyl proteases, cathepsins D and E (Figure 7). Cathepsins D and E are potential drug targets as their overexpressions have been observed in various cancer forms, such as pancreatic ductal adenoma, cervical adenocarcinoma, lung carcinoma, and gastric adenocarcinoma [100]. The linear decadepsipeptides, grassystatins A-F (e.g., **39**) were isolated from *Lyngbya confervoides* and VPG 14-61 collected at Grassy Key, Florida and Cetti Bay, Guam, respectively [101,102]. These statine unit-containing molecules were initially isolated from a screening program by evaluating the inhibitory activities of natural products against 59 proteases. Grassystatins A (**39**) and B displayed potent inhibitory activity against cathepsins D and E with IC_50_ values averaging at 16.9 nM and 0.62 nM, respectively. In addition, grassystatin A was able to reduce antigen presentation by dendritic cells [101]. The total synthesis of grassystatin A was recently accomplished by Yang and co-workers and pharmacological studies revealed that the inhibition of cathepsin E by the molecule did not impact ovalbumin antigen processing and peptide presentation [103]. In addition, grassystatin F was shown to inhibit the cleavage of cystatin C and plasminogen activator inhibitor type-1 (PAI-1) via its inhibition on cathepsin D. Cystatin C and PAI-1 are involved in the downstream activation of cysteine cathepsins and tissue plasminogen activator (tPA), which would lead to the migration of highly aggressive triple negative breast cancer cells (MDA-MD-231) [102].

Tasiamides are grassystatin-related molecules found to possess potent cathepsins D and E inhibitory activity. In particular, tasiamides B (**41**) and F (**40**) (Figure 7) displayed potent activity against cathepsins D and E, with IC_50_ values of 50 nM and 9.0 nM for **41** and 57 nM and 23 nM for **40**, respectively [104]. When tested against BACE1 (β-site Amyloid precursor protein Cleaving Enzyme type 1), an enzyme implicated in Alzheimer’s disease, compound **40** was about 12- to 30-fold less active as an BACE1 inhibitor compared to cathepsins D and E [104]. A number of synthetic tasiamide B analogues, such as compounds **42** and **43** (Figure 7), have been developed as selective inhibitors of these aspartic proteases [105,106,107]. Compound **42** was found to be highly selectivity for cathepsin D with 576-fold over cathepsin E and 554-fold over BACE1 [106]. In another study by Li et al., synthetic compound **43** (Figure 6) displayed significant selective inhibitory activity against cathepsin D with IC_50_ of 3.29 nM over cathepsin E (72-fold) and BACE1 (295-fold) [107]. These results could path the way for the development of highly selective cathepsin D inhibitors.

Symplocin A (44) (Figure 7) is another highly potent cathepsin E inhibitor isolated from the Bahamian cyanobacterium, *Symploca* sp. [108]. Symplocin A is structurally related to grassypeptins in having a statine-unit. In addition to Marfey’s method, a new strategy using 2-naphthacyl esters of *N*,*N*-dimethylamino and 2-hydroxy acids were employed for absolute stereochemistry determination of this molecule. Symplocin A is a potent inhibitor of cathepsin E with IC_50_ value of 300 pM, which is comparable to that of pepstatin, a known inhibitor of aspartyl proteases. Taken together, the biological data of these series of statine-containing compounds, including grassystatins, tasiamides, and symplocin A, showed that selectivity can be tuned and these structural scaffolds can serve as a starting point for development of selective aspartic protease inhibitors.

#### 2.3.4. β-Secretase 1 (BACE1) Inhibitors

##### Tasiamide B

**Figure 8:**
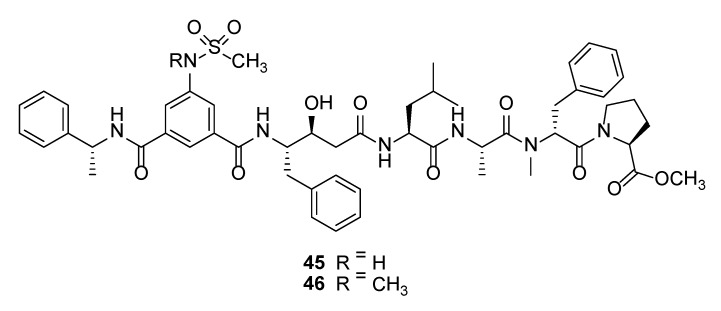
Tasiamide B-related compounds.

Tasiamide B (**41**) (Figure 7) is a statine-containing linear depsipeptide shown to inhibit the aspartic protease, BACE1, with an IC_50_ of 0.19 µM [105,109]. BACE1, also known as β-secretase 1, is a potential drug target for the treatment of Alzheimer’s disease (AD) due to its involvement in the abnormal production of β-amyloid plaques in AD patients [110,111]. The total synthesis of tasiamide B led to a revision in stereochemistry on the structure of the original reported molecule [112]. Several synthetic tasiamide B-analogues have been synthesized with superior inhibitory activities against BACE1. For instance, hybrid molecules containing structural features of tasiamide B and sulfonamide-containing isophthalic acid unit, such as compounds **45** and **46** (Figure 8) showed potent inhibition of BACE1 with IC_50_ of 128 nM and 57.2 nM, respectively [105]. Moreover, these synthetic molecules showed selectivity for BACE1 over γ-secretase and compound **38** exhibited in vivo activity by reducing levels of amyloid β-peptide in brain of rodent [105]. Recent SAR studies based on 19 synthetic analogues of tasiamide B revealed the importance of the hydrophobic substituents, valine, leucine, alanine, and phenylalanine, for inhibitory activity against BACE1 [113].

### 2.4. Interference of the Actin and Microtubule Filaments

#### Dolastatins 10/15

**Figure 9:**
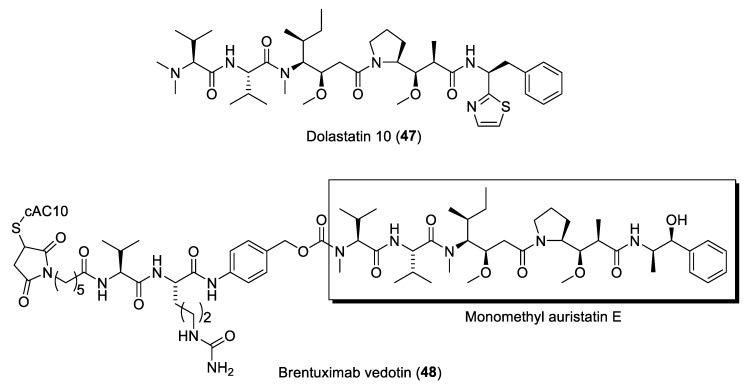
Dolastatin 10 and brentuximab vedotin.

One of the earliest examples of potent microtubule inhibitors reported from marine cyanobacteria are the dolastatin class of molecules, including dolastatins 10 (**47**) and 15 (Figure 9) [114]. The dolastatins were originally isolated from the Indian Ocean sea hare, *Dolabella auricularia*. However, these cytotoxic compounds are now known to be produced by marine cyanobacteria due to the isolation of a number of dolastatin-related molecules from these microbes [115]. Dolastatins 10, 15 and their synthetic analogues, including the auristatins, have undergone a number of clinical trials as anticancer agents [11]. It was eventually realized that the auristatins are of great value as payloads in antibody drug conjugates (ADCs) formulation [116]. This discovery led to the FDA-approved ADC brentuximab vedotin (**48**) (Figure 9) in 2011 for the treatment of elapsed or refractory Hodgkin’s lymphoma as well as systemic anaplastic large cell lymphoma [117]. Brentuximab vedotin targets CD30 of tumor cells and selectively delivers monomethyl auristatin E (MMAE) into the cells and induces cancer cell apoptosis. Numerous modifications of the N- and C-terminal groups in auristatins have been synthesized. A review on the design of new auristatins and SAR analysis has been published [118]. Currently, more than 16 ADCs, incorporating auristatins as payloads, are currently in clinical trials for cancer therapy [119].

### 2.5. Sec61 Protein Translocation Channels

#### 2.5.1. Apratoxin A

**Figure 10:**
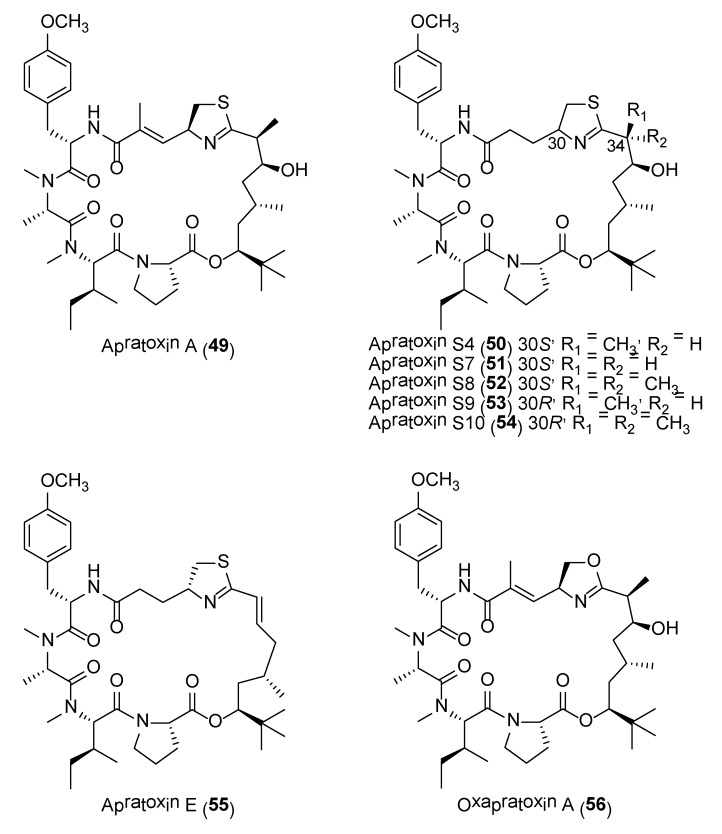
Apratoxin A and related compounds.

The apratoxins are a novel class of potent cytotoxic cyclodepsipeptides reported from several *Lyngbya* sp. strains collected from various locations. They possess significant biological activities in the nanomolar range when tested against a panel of cancer cell lines, including HT29, HeLa, and U2OS. To date, a total of nine apratoxin-related compounds have been reported with apratoxin A (**49**) (Figure 10) being the most cytotoxic [120]. The biosynthetic gene cluster of apratoxin A was identified via a single-cell genome-amplification approach and reveals a PKS-type loading module and nine extension modules, including four polyketide synthases (PKS) and five nonribosomal peptide synthetases (NRPS) [121]. It was recently reported that the *tert*-butyl group in apratoxin A is formed as a pivaloyl acyl carrier protein by AprA, the PKS loading module of the apratoxin A biosynthetic pathway [122]. Due to the significant potency of apratoxins, a number of synthetic efforts on the generation of potent synthetic analogues have been achieved. For instance, synthetic analogues, such as apratoxin S4 (**50**) and apratoxins S7 (**51**) to S9 (**53**), with improved antitumor activity, inhibitory secretion of the angiogenic vascular endothelial growth factor –A (VEGF-A) and tolerability in human HCT116 xenograft mouse model at subnanomolar IC_50_ concentrations have been reported(Table 4) (Figure 10) [123,124]. The structures of these synthetic molecules are based on the structural features of apratoxins A (**49**) and E (**55**), giving rise to the apratoxin A/E hybrid structure, differing in various degrees of methylation at C34 as well as epimeric configuration at C30. From the biological data, it was revealed that the configuration of C-34 is independent of the cytotoxic activity (Table 4).

Further studies on apratoxin S4 (**50**), revealed the molecule to possess potent antiangiogenic activity by inhibiting the activation of retinal endothelial cells and pericytes through mediating multiple angiogenic pathways [125]. This finding could path the way for the development of apratoxin S4 as a potential cure for prevention or treatment of vision loss. A scalable synthesis of a stable analogue, apratoxin S10 (**54**) (Figure 10), was shown to potently inhibit angiogenesis in vitro and growth of cancer cells from vascularized tumors [126]. A recent study revealed another synthetic analogue, apratoxin S10, as a potential anti-pancreatic cancer agent [127]. The study showed that the molecule inhibited the growth of established and patient-derived primary pancreatic cancer cells. The growth inhibitory activity of apratoxin S10 on pancreatic cancer cells was due to the downregulation of multiple receptor tyrosine kinases as well as inhibition of growth factor and cytokine secretion [127].

A series of mechanistic studies on apratoxin A (**49**) revealed its ability to interfere with specific cellular signaling pathways as well as protein interactions involved with the formation and maintenance of cancer cells. Using functional genomics approach, apratoxin A was found to exert its antiproliferative property through induction of G1 cell cycle arrest and apoptosis by antagonism of the fibroblast growth factor (FGF) signaling via Signal Transducer and Activator of Transcription 3 (STAT3) [128]. In another study based on a synthetic oxazoline analog of apratoxin A, it was found that 56 (Figure 10) stabilizes the Hsp90 (heat shock protein 90) client proteins-Hsc70 /Hsp70 interaction, thereby inhibiting the function of Hsp90 [129]. The inhibition of Hsp90 resulted in the promotion of Hsp90 client proteins degradation via chaperone-mediated autophagy. Apratoxin A was also found to inhibit the secretory pathway by preventing cotranslational translocation of newly synthesized secretory and membrane proteins into the ER [130].

The specific mode of action of apratoxin A was eventually elucidated in two studies conducted by Paatero et al. and Huang et al. [131,132]. Apratoxin A kills cancer cells by directly blocking the Sec61 protein translocation channel [131,132]. Specifically, apratoxin A prevents protein translocation into the ER by direct binding with the central subunit of the protein translocation channel, Sec61α [131]. Binding of apratoxin A on the luminal end of the Sec61 lateral gate resulted in blocking the biogenesis of a range of Sec61 clients [131]. In addition, pathologic studies revealed apratoxin A to target pancreas, resulting in severe pancreatic atrophy in apratoxin A-treated animals [132]. The importance of Sec61 as a potential drug target is a promising concept for anticancer therapy. Recently, it was reported that the function of human epidermal growth factor receptor 3 (HER3), implicated in several cancer types, can be inhibited through direct binding of Sec61 with substrate-specific Sec61 inhibitor, cotransin [133].

#### 2.5.2. Coibamide A

**Figure 11:**
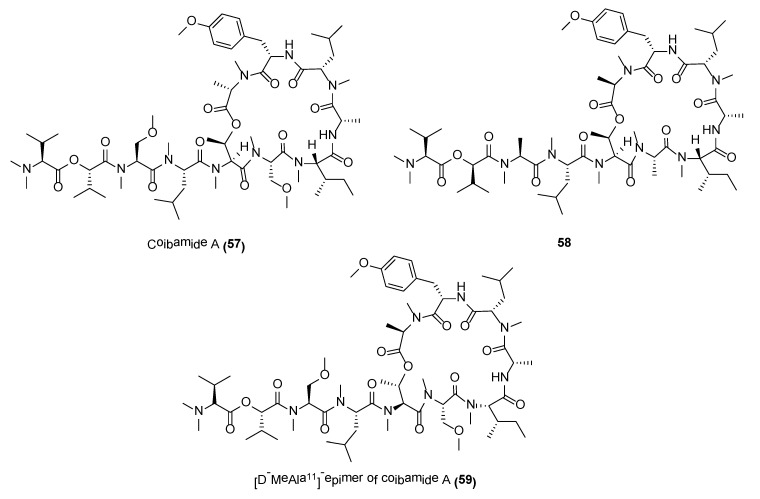
Coibamide A and related compounds.

Coibamide A (57) (Figure 11) is a structurally novel lariat-type cyclic depsipeptide, with potent antiproliferative properties, reported from the Panamanian marine cyanobacterium, *Leptolyngbya* sp. [134]. The complete structure of coibamide A (**57**) was deduced by various 2D NMR spectroscopic experiments, including COSY, TOCSY, multiplicity-edited HSQS, HSQC-TOCSY, HMBC, H2BC, 1H-15N gHMBC, and ROESY as well as mass spectroscopic data. This molecule possesses a high degree of N-methylation with eight out of 11 residues being N-methylated. More importantly, coibamide A displayed potent cytotoxicity against NCI-H460 lung cancer cells and mouse neuro-2a cells, with LC_50_s reported to be less than 23 nM. The compound was evaluated in the NCI’s panel of 60 cancer cell lines and it exhibited significant activities against MDA-MB-231, LOX IMVI, HL-60(TB), and SNB-75 at IC_50_ values of 2.8 nM, 7.4 nM, 7.4 nM and 7.6 nM, respectively [134]. Total synthesis of coibamide A was accomplished using solid-phase peptide strategy by several synthetic groups. These synthetic efforts led to the revision on the stereochemical assignment on the original reported molecule as well as generation of potent analogues for SAR studies [135,136,137]. Several synthetic analogues, such as **58** and **59** (Figure 11), showed either similar inhibitory activity or increased cytotoxicity as compared to the natural product [136,137]. A number of pharmacological studies have also been conducted on coibamide A [138,139,140]. A recent study, using a synthetic coibamide A photoaffinity probe, showd that coibamide A directly targets the Sec61α subunit of the trimeric Sec61translocon. The binding of the molecule to Sec61 resulted in broad substrate-nonselective inhibition of ER protein import and conferred potent cytotoxicity against specific cancer cell lines [141].

### 2.6. Interference of Inflammatory Pathways

#### Honaucins

**Figure 12:**
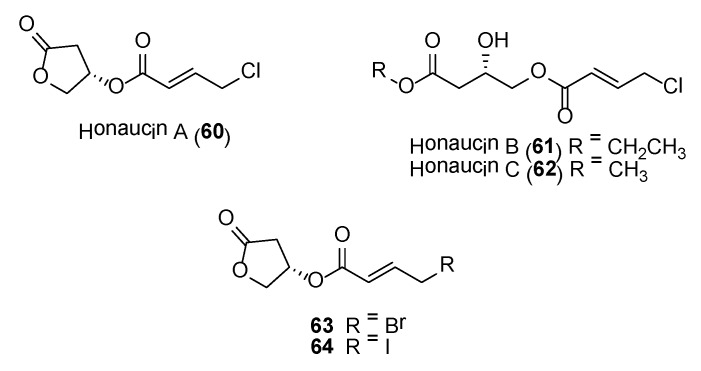
Honaucins and related compounds.

Honaucins A (**60**)–C (**62**) (Figure 12) are potent anti-inflammatory and bacterial quorum sensing (QS) inhibitory molecules isolated from a collection of *Leptolyngbya crossbyana* found overgrowing on corals in Hawaii [142]. The structure of the major compound, honaucin A, consists of a unit each of (*S*)-3-hydroxy-γ-butyrolactone and 4-chlorocrotonic acid, connected via an ester linkage. These molecules showed inhibition of bioluminescence in *Vibrio harveyi* BB120 and lipopolysaccharide-stimulated nitric oxide production in the murine macrophage cell line RAW264.7 [142]. Additional pharmacological studies performed on honaucin A showed that the molecule’s ability to attenuate inflammation via activation of the Nrf2-ARE pathway [143]. Synthetic analogues, based on honaucin A, revealed that the halogen atom at the 4-position of the crotonoic acid moiety was essential for both anti-inflammatory and QS inhibitory activities.

Two synthetic analogues, 63 and 64 (Figure 12), were found to possess enhanced anti-inflammatory and QS inhibitory properties when compared to the natural products [142]. Subsequent pharmacological studies conducted on the bromo-analogue of honaucin A, compound 63, showed that it inhibited osteoclastogenesis from macrophage cell line in vitro and reduced the RANKL-induced expression of osteoclast-associated genes, such as MMP9, Cath K, GAB2, C-MYC, C-SRC, MITF, PU 1 and DC-STAMP [144]. These results showed that compound 63 could have potential as a therapeutic drug for treatment of disorders associated with bone loss.

## 3. Conclusions

This review covers a range of unique marine cyanobacterial natural products as well as their synthetic analogues having potent biological activities, including anticancer and antiinfective properties. A majority of these compounds are nitrogen-containing and products of the modular PKS-NRPS metabolic pathways. Due to their exquisite interference against clinically relevant drug targets, such as proteasomes, proteases and microtubule filaments, they represent important lead compounds for further development into therapeutic drugs.

A number of noteworthy drug leads include largazole, apratoxin A, carmaphycins, gallinamide A and the dolastatin class of molecules. Drug candidates, such as the largazole-based synthetic analogue, OKI-179, and apratoxin S4 have been identified from synthetic analogues of these natural drug leads and are currently undergoing or being considered for clinical testing. In addition, a high number of auristatin-based compounds, formulated as ADCs, are undergoing clinical testing as anticancer agents. Unique structural features attributed to marine cyanobacterial compounds could account for their high potency. These include high degree of N-methylation (e.g., coibamide A), macrocycles with incorporation of polyketide moiety (e.g., apratoxins), presence of heterocycles, such as thiazole, thiazoline, oxazole oxazoline and methoxypyrrolinone (e.g., apratoxins, gallinamide A, dolastatins 10 and 15), incorporation of D- and non-proteinogenic amino acids (e.g., 2-aminobutenoic acid unit in lyngbyastatin 7) as well as peptide acylation (e.g., largazole). These structural features contribute to compound permeability and adoption of desired conformation for interaction of cellular drug targets. Moreover, combination of these structural motifs expands the chemical space of marine cyanobacterial natural products.

**Figure 13:**
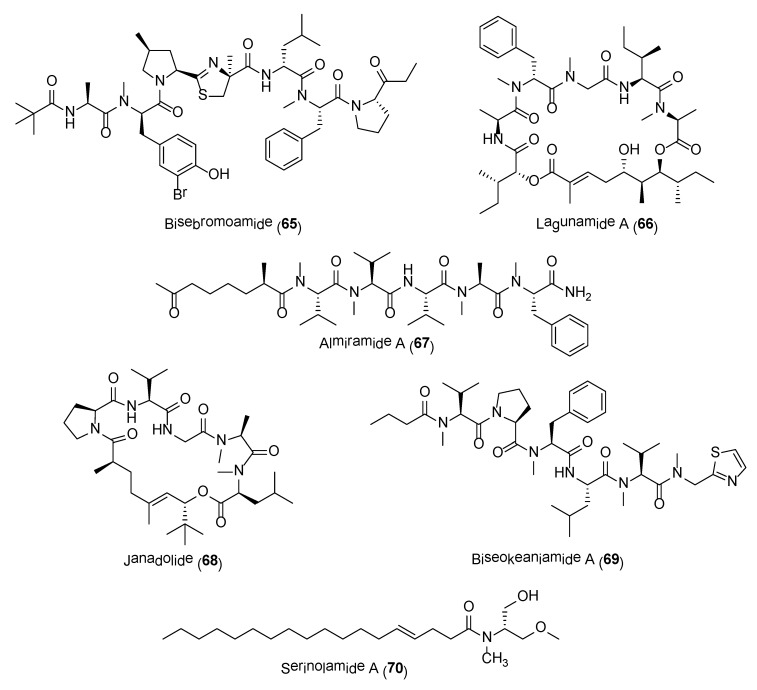
Other potent marine cyanobacteria compounds.

The range of molecules presented in this review is by no means exhaustive, as there are numerous potent cyanobacterial compounds reported in the literature and are beyond the scope of this review. These compounds include the highly cytotoxic compounds, bisebromoamide (**65**) and aurilide-class of compounds (e.g., **66**), antiinfective molecules, almiramides (e.g., **67**) and janadolide (**68**), anti-inflammatory molecule, biseokeaniamide A (**69**) as well as cannabimimetics/CNS modulatory agents, such as mooreamide A and serinolamides (e.g., **70**) (Figure 13) [145,146,147,148,149,150,151,152,153]. Further biological evaluation and synthesis of analogues have also been initiated for some of these compounds [154,155,156,157,158,159]. The search for novel bioactive cyanobacterial compounds has also been expedited with recent advances in genomic and metabolomics techniques, e.g., the integrated use of mass spectrometric based molecular networking and genomic approaches [160,161]. It is without a doubt that these prokaryotic marine cyanobacteria will continue to provide essential drug leads in drug discovery and development efforts.

## Figures and Tables

**Table 1:** Summary of pharmacological studies on largazole (**1**).

Disease/Target	Significant Biological Activity	Reference
Angiogenesis-associated diseases	Topical application of the compound attenuated alkali-induced corneal neovascularization in mouse model. Down-regulated the expression of the pro-angiogenic factors and up-regulated the expression of the anti-angiogenic factors *in vivo*.	[31]
Lung cancer	Potently inhibits the proliferation and clonogenic activity in lung cancer cells. Arrests cell cycle at G1 phase and up-regulates the expression of cyclin-dependent kinase inhibitor p21 in lung cancer cells. An E2F1-targeting cell cycle inhibitor, which is overexpressed in lung cancer tumor.	[32]
Rheumatoid arthritis	Activates p38 and Akt pathways and increase expression of HDAC6 by more than 200% in rheumatoid arthritis synovial fibroblasts. Increases HDAC6 expression, which led to enhancement of the detrimental effects of TNF-α in RA synovial fibroblasts.	[33]
Liver fibrosis	Reduces liver fibrosis and angiogenesis by inhibition of transforming growth factor-b as well as vascular endothelial growth factor signalling.	[34]
Breast cancer	Cooperates with dexamethasone to induce localization of E-cadherin to the plasma membrane in breast cancers as well as to suppress in vitro cellular invasion of cancer cells.	[35]
Protein ubiquitination	Largazole and its ester and ketone analogs selectively inhibit ubiquitin conjugation to p27^Kip1^ and TRF1 *in vitro*.	[36]
Epstein-Barr virus (EBV)-associated lymphomas	Induces expression of EBV lytic-phase gene and sensitize lymphoma cells to nucleoside antiviral drugs.	[37]
Bone-related disorders	Exhibits in vitro and in vivo osteogenic activity via increased expression of Runx2 (runt-related transcription factor 2) and BMPs (bone morphogenetic proteins). Induces expression of alkaline phosphatase (ALP) and osteopontin (OPN).	[38]
Colon cancer	Strongly stimulated histone hyperacetylation in tumor in vivo by using a human HCT116 xenograft mouse model. Efficacy in inhibiting tumor growth and induced apoptosis in the tumor. Regulates transcription of genes involved in the induction of cell cycle arrest and apoptosis.	[39]
Oncogenic super-enhancers	Decreases RNA polymerase II accumulation at super-enhancers (SEs) and preferentially suppresses SE-driven transcripts associated with oncogenic activities in transformed cells.	[40]
Antiretroviral therapy	Combination of largazole and bryostatin analogues are potent activators of latent HIV without global T-cell activation within resting CD4+ T-cells.	[41]

**Table 2:** HDAC inhibitory activities of largazole-based synthetic analogues **3** to **6** (IC_50_ nM).

HDAC	Compound			
	3	4	5	6
1	2.20	21.0	1.0	40.0
2	4.42	28.0	1.9	90.0
3	2.31	27.0	1.5	43.0
5				>10,000
6	35.16	13,000	0.24	2800
8	101.8			9600
10				3.6

**Table 3:** Inhibition of falcipains 2, 3 and various strains of *P. falciparum* by gallinamide A analogues **32**–**36** (IC_50_ nM).

Compound	Falcipain		*P. falciparum*		
	2	3	3D7	Dd2	W2
**32**	5.25	81.4	20.0	67.0	
**33**	12.0	66.7	9.7	29.0	
**34**	31.0	117.0	26.0		28.0
**35**	29.0	79.0	42.0		49.0
**36**	33.0	112.0	1.0		4.0

**Table 4:** Activities of apratoxin-related analogues, apratoxins S4 (**50**), S7 (**51**)–S9 (**53**) on viability of HCT116 cell and secretion of VEGF-A (IC_50_ nM).

Apratoxin	HCT116	VEGF-A Secretion
S4 (**50**)	1.43	0.32
S7 (**51**)	1.25	0.30
S8 (**52**)	1.99	0.47
S9 (**53**)	0.69	0.12
